# Reduction Mammaplasty: What Cup Size Will I Be?

**DOI:** 10.1097/GOX.0000000000002273

**Published:** 2019-06-14

**Authors:** Marion Chan, Sarah Lonie, Sean Mackay, Kirstie MacGill

**Affiliations:** From the * Plastic Surgery Unit, Eastern Health, Melbourne, VIC, Australia; † Eastern Health Surgical Research Group, Department of Surgery, Monash University, Eastern Health, Melbourne, VIC, Australia.

## Abstract

**Background:**

Predicting cup size after reduction mammaplasty is a challenge well recognized by plastic surgeons. This study presents a method whereby the weight of tissue to be excised can be predicted on the basis of the initial and desired cup size.

**Methods:**

Breast density was calculated from resection specimens. Cup volumes of a specific range of bra style were then measured by filling the bra cups with modeling clay on a mannequin and the volume measured via water displacement. These data were then correlated to breast tissue volume and weight.

**Results:**

The average breast tissue density calculated was 0.98 g/ml (SD = 0.05). Bra cup volume measurements showed a steady progression according to both cup and band sizes. A table was constructed to predict the weight of tissue required for excision to achieve the desired change in cup size.

**Conclusion:**

These results can assist plastic surgeons in predicting the amount of breast tissue to excise to achieve a given cup size. A secondary use of these results is a breast volume guide for implant planning.

## BACKGROUND

A challenge for plastic surgeons performing reduction mammoplasty is to predict the volume of tissue to remove to achieve the patient’s desired outcome. Previous methods attempted to reliably estimate breast size and predict resection volumes for breast reduction procedures have included water displacement, casting, and more recently the development of 3-dimensional imaging systems.^1–5^ However, due to factors such as cost and feasibility, none of these methods have become established in the clinical setting.

Regnault and Daniel^6^ initially proposed a formula for estimating the resection weight of breast tissue during reduction mammaplasty to achieve a desired cup size. This technique and the data were subsequently validated and improved upon by Turner and Dujon.^1^ Descamps et al^7^ described another formula to determine resection weights by correlating the following 4 variables: notch to nipple distance, nipple to inframammary crease distance, body mass index, and age. Unfortunately, these methods relied heavily on metric measurements of the female breast and the traditional brassiere measuring system, the latter which was shown by Turner and Dujon^1^ to result in the underestimation of breast reduction weight. Moreover, Pecter^8^ noted that in his method of determining cup size, the measured band size on a woman would not match the actual circumference of a manufactured bra. An example described by the author is a woman with an underbust chest circumference of 30 inches, who would wear a bra with a band size of 36. However, measurement of size 36 bras from 3 different manufacturers indicated an average circumference of only 27 inches. It is the elastic property in the band that allows the bra to stretch to the 30 inches necessary to fit comfortably around the woman’s chest.

Developing a better technique to estimate resection volumes would benefit plastic surgery trainees and junior surgeons in performing such a procedure more accurately and also aid in satisfying patient curiosity during the initial consultation with respect to how much tissue might be excised. Moreover, in some parts of the world, estimated resection weights must be obtained by insurance companies before a reduction procedure in order for claims to be processed.

Traditionally, bra sizes as labeled by the manufacturers are determined by using the conventional brassiere sizing system, which takes into account band size and bust circumference. Band size is measured by the circumference of the chest immediately below the breasts, and bust circumference is measured around the fullest part of the breasts. The planned postoperative cup size is determined by patient preference, aesthetic considerations such as the balance among the patient’s height, hips, and bust, and surgical considerations such as pedicle size and skin flap thickness. It should be noted that most, if not all, breast reduction techniques affect a patient’s cup size only and do not affect band size.

The aims of the study were to develop a method to predict resection weights for reduction mammaplasty procedures to achieve desired cup sizes and create a useful format to use this information as a guide in chart format. To achieve this, we needed to establish first that breast weight can be attributed to volume and second volume measurements of cup sizes.

## METHODS

This study was conducted at Box Hill Hospital, Eastern Health, Melbourne, VIC, Australia.

### Ensuring Breast Weight Can Be Attributed to Breast Volume

Although the volume (in milliliters) of a reduction specimen is a major factor in determining the final outcome of a reduction procedure, it is much more common for surgeons to obtain measurements in weight (usually in grams). Thus, to present any predicted resection amounts in a similar way (ie, grams), it was necessary to correlate breast weight (grams) to breast volume (in milliliters). As a result, breast density measurements were taken as a pilot study (stage I—see below) before commencement of stage II—measuring bra cup volumes.

### Stage I—Measuring Breast Density

Data were collected from breast tissue specimens excised from mastectomies, wide local excisions, and breast reduction surgeries over the course of a 6-month period. Each specimen was weighed intraoperatively with a set of digital scales and volume measured via water displacement with a 1900cc graduated Receptal suction canister (Hospira, Inc. Lake Forest, Ill., USA). Each excised breast tissue specimen was completely submerged in the canister containing water at room temperature upon a level surface. Compression of the specimen was avoided, and the increase in volume was read from the scale on the canister to an accuracy of 25 ml. Small volume tissue specimens were excluded from the study to maintain accuracy. The density of each breast specimen was then calculated by dividing mass (grams) by volume (milliliters).

### Canister Calibration

The Receptal suction canister is manufactured for use with a plastic liner on the interior surface, and this liner was not used for measurements in this study. Before commencing measurements in stages I and II, the Receptal suction canister was calibrated without the liner by filling the canister with a volume of water as specified by the scale as marked on the canister and then cross-checking the accuracy of the markings by weighing the volume of water with a set of digital scales. A linear relationship was found from this calibration exercise, and a graph was plotted to show this. A line of best fit was drawn through the plotted values on the graph, and a linear equation was obtained for this line of best fit (*R*^2^ = 0.99945) by using Microsoft Office Excel 2007 [*y* = 1.0663*x*, where *y* = weight of fluid in canister (grams) and *x* = volume of fluid in canister (milliliters)]. All measurements recorded from the study were subsequently adjusted by using this equation.

### Stage II—Measuring Bra Cup Volumes

We standardized our bra cup measurements by limiting measurements to bras provided to us by one style from one single company only. Twenty-two bras (Symphony W style number 10075626) were provided by Triumph International Australia. This style of bra was chosen for a number of reasons. First, it is an enclosed style that leaves less scope for variation in accurately filling the cups (when compared with a lower-cut style, which may allow a variable amount of breast tissue to bulge over the top of each cup); second, availability of a comprehensive range of band and cup sizes (whereas some styles are specifically made for smaller or larger breasted women and do not cover a large size range); and third, the availability of this brand in Europe, Asia, Africa, America, and Australasia to aid with generalizability of this study.

**Fig. 1.**
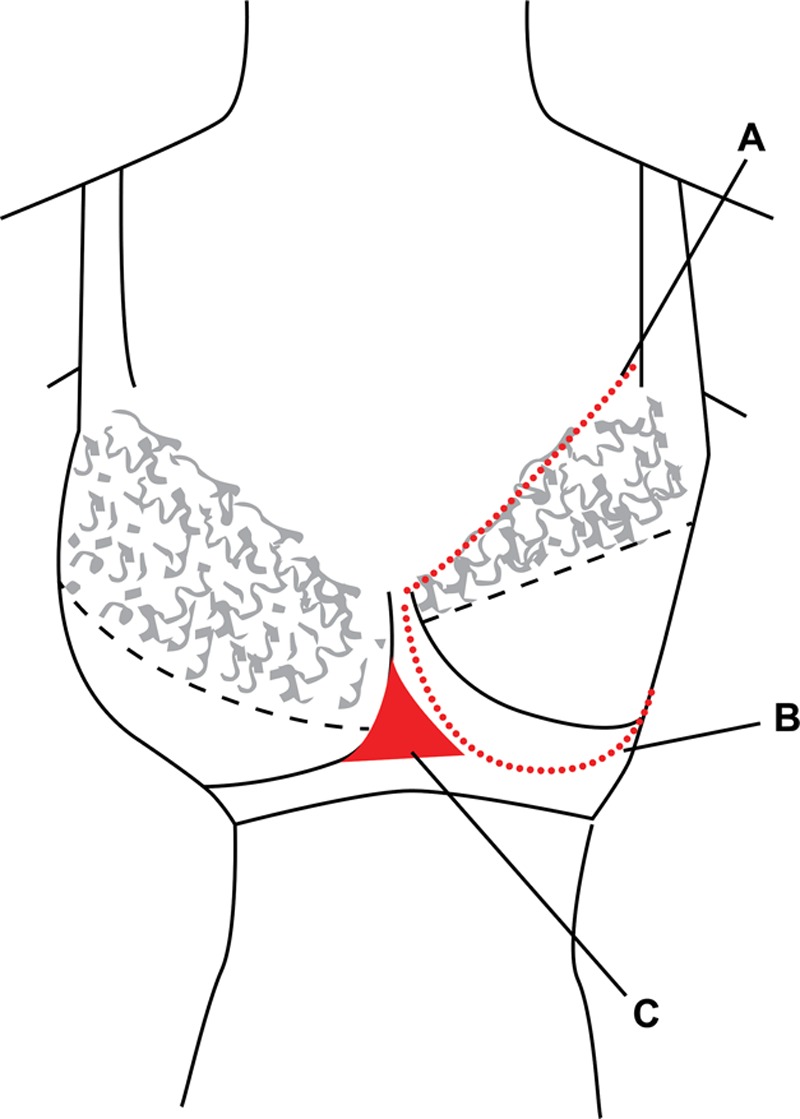
A bra fitted to an adjustable mannequin and filled with modeling clay on the right side. A, Line of stitching across the top of the bra. B, Underbust wire support. C, Midpoint of the front of the bra.

With the assistance of a professional bra fitter from Triumph©, each bra was fitted onto a mannequin with an adjustable chest circumference and each right-sided cup filled with modeling clay (Fig. 1). Parameters for filling the bra were of similar principles to those professional bra fitters would regularly use as a guide: (1) the underbust wire support of the bra (inclusive of the anterior midpoint of the bra) must remain in contact with the chest wall of the mannequin at all times; (2) the filling of clay should not extend posteriorly beyond the anterior axillary line of the mannequin or beyond the line of stitching across the top of the bra; and (3) the line of stitching across the top of the bra should lie reasonably close to the chest wall of the mannequin along its entire length. The resultant mass of modeling clay fitted to each bra cup size was then weighed, and the volume measured by water displacement, using the same technique as described earlier for the breast specimens.

### Establishing Bra Cup (Volume) Measurements

The volume of reduction required to achieve a desired cup size was considered as equivalent to the difference between the preoperative and postoperative cup sizes. This was plotted in the table form of volume differences in cup size for a given chest circumference to determine resection volume.

### Data Analysis

Data were analyzed using SPSS Statistics 17.0 (©IBM Corporation 2010, Somers, N.Y., USA). Data on various band/cup volumes were tabulated manually.

## RESULTS

### Breast Density

**Fig. 2.**
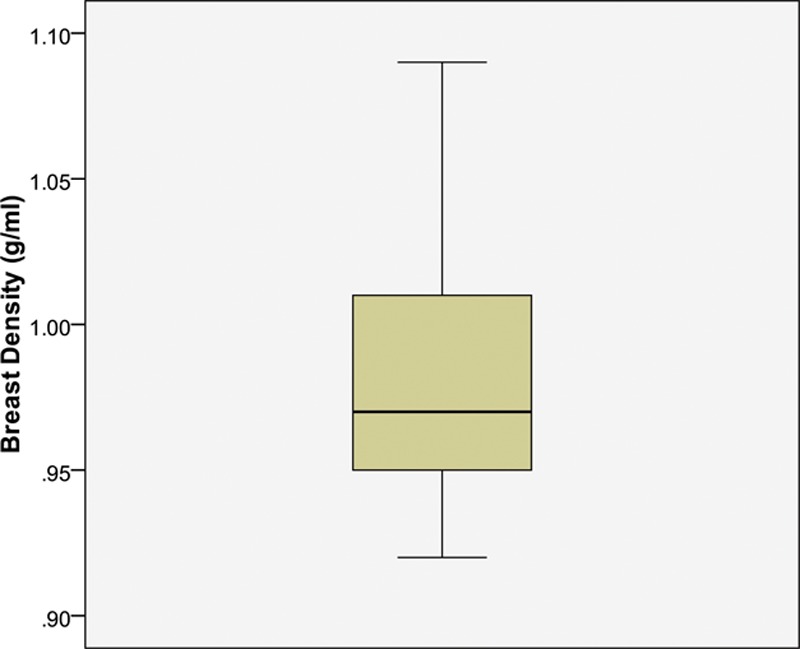
Distribution of breast density (g/ml) values calculated from stage I of study. The whiskers represent the full range of the data, the box represents the interquartile range, and the dark bar represents the median point.

Fifteen breast specimens were obtained in stage I and consisted of 9 unilateral mastectomies (4 of these patients also underwent axillary clearances), 2 wide local excisions, and 8 specimens from 4 cases of bilateral reduction mammaplasty. To prevent double measuring, tissue specimens from only the right breasts were considered from reduction mammaplasties. The resultant weight of breast tissue excised for each case was divided by its respective volume displacement measured to calculate density (grams per milliliters) (Fig. 2). The average breast tissue density calculated was 0.98 g/ml (SD 0.05). The minimum density calculated was 0.92 g/ml, with a maximum value of 1.09 g/ml.

### Bra Cup Volumes

**Fig. 3.**
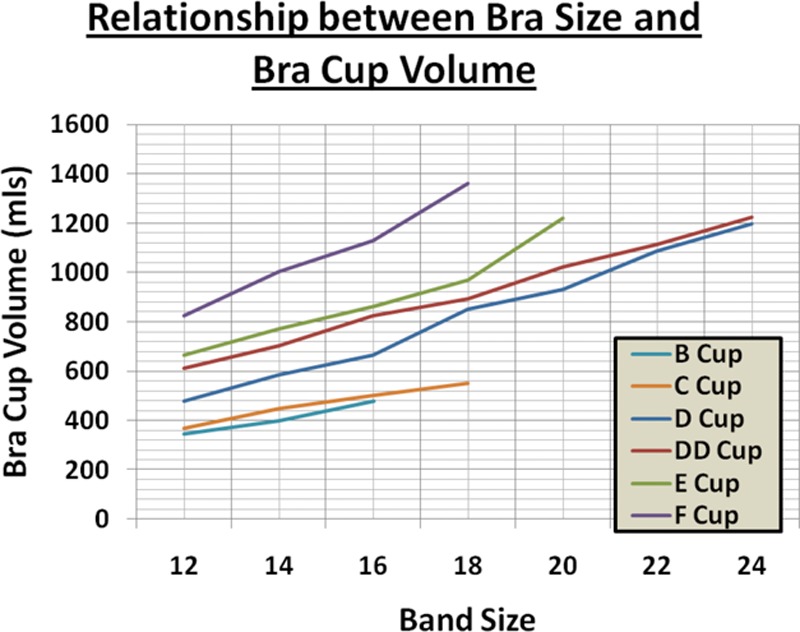
Graph showing distribution of bra cup volume across different bra sizes.

Bra cup volume measurements were plotted (Fig. 3) and show a steady progression according to both cup and band sizes.

**Fig. 4.**
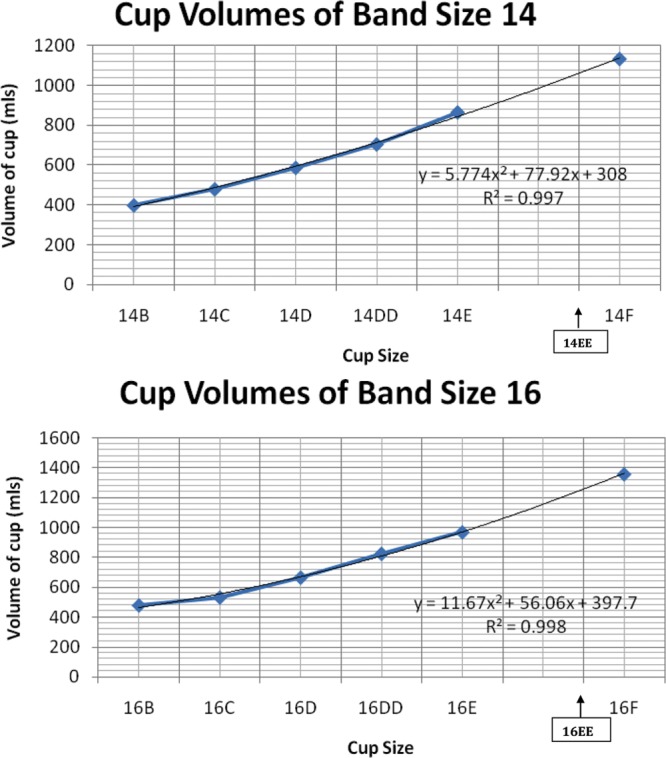
Graph showing distribution of bra cup volume across (A) band size 14 and (B) band size 16.

An interesting observation from this exercise was that the volumes of larger cup sizes (such as E and F) increased in a rapid manner with small increases in band sizes beyond 14 and 16. When an extra cup size (EE) was added onto the graph, however (Fig. 4), we found that the volume curve increased much more steadily. Although an EE cup size was not available in this style, some manufacturers do provide this extra cup size.

### Control

Techniques were also used to control bra fitting and clay filling errors and inconsistencies. After fitting all 22 bras to the mannequin with modeling clay, the professional bra fitter was then asked to refit and refill 5 of the 22 bras chosen to encompass the range of volumes encountered. The volumes recorded for these 5 bras and the amount in which they differ from their initial measurements are shown in Table 1. As expected, a greater percentage of error was noted for the smaller volumes.

**TABLE 1.**
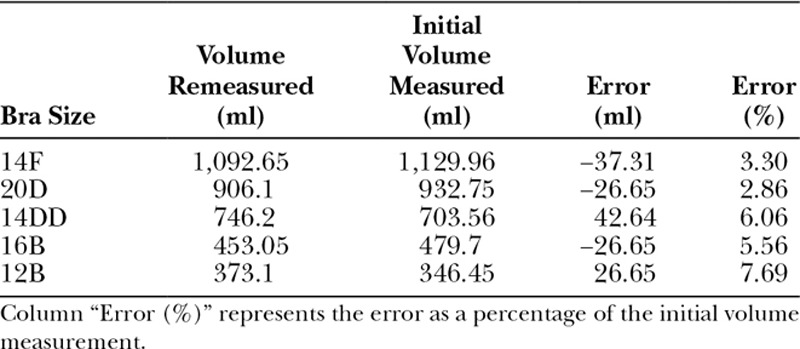
Volumes of the 5 Bras That Were Refitted and Refilled

### Correlation of Stages I and II Data

**Fig. 5.**
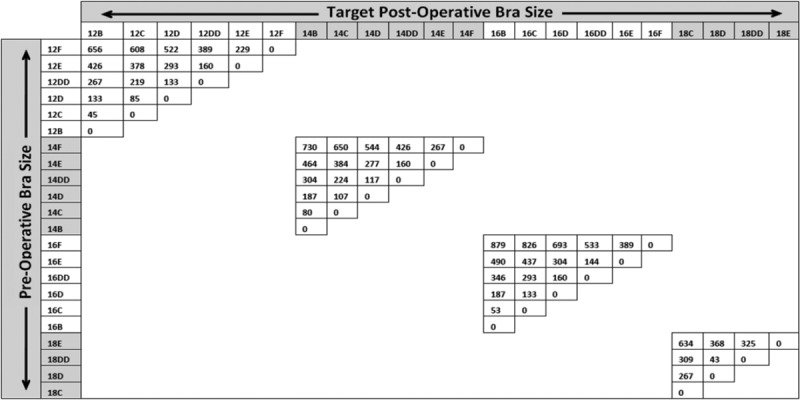
Volume changes required for change in cup size (band sizes 12–18).

As stated earlier, breast tissue weight is more easily measured in the operating theater in comparison with breast tissue volume. From the data obtained in stage I, it was shown that breast density is of a value 0.98 g/ml, with an SD of 5%. Hence, the bra cup volumes measured from stage II of the study can reasonably be correlated to weight of breast tissue. Figure 5 was then constructed from these data to predict the weight of tissue required for excision to achieve the desired change in cup size.

## DISCUSSION

We successfully determined cup size and correlation with volumes and tabulated resection volumes in reduction mammoplasty to obtain the correct change in cup size. The volume of the patient’s breasts can be estimated according to the data in Figures 3 and 5. During the preoperative assessment, the suggested or desired cup size could also be fitted to a mannequin to illustrate the expected result postsurgery. The surgeon may then consult the data in Figure 5 as a guide to predict the amount of breast tissue to excise. A secondary use of these results is estimating cup size changes for implant procedures (Fig. 5) or breast volume for a given band and cup size (Fig. 3) to plan for mastectomy and implant-based reconstruction.

Our finding that for a given cup size, volume increases with band size supports the paradigm in which band size is regarded as a variable measurement, which ultimately determines cup size.^9^ In general, similar cup volumes may be obtained by decreasing the band size as the cup size increases and vice-versa by increasing the band size as the cup size.^10^ This technique is often used by commercial bra fitters. For example, a woman who would normally wear a size of 14B bra can also be fitted with a size of 12C bra as both bras are of similar volume (provided that the smaller band is not too tight). Obviously, these results are only a guide and the treating surgeon will also consider aesthetic factors such as the patient’s height, overall build, and the proportions of the bust and hips.

A limitation in this study and calculating reduction volumes may be that patients individually cannot be relied upon to wear correctly fitted bras and the same size bra made by different commercial companies may not necessarily be of the same volume. The topic of wearing “wrong sized bras” has attracted much attention, both in the popular media and in the medical literature, especially in relation to aesthetic breast surgery.^8,11,12^ Women may wear a bra sized incorrectly according to the judgment of a professional bra fitters: supporting wires that compress or cut into the breast; or where the anterior midpoint of the bra is not lying flat against the chest; or where the back horizontal strap is running too high or too low on the back to provide adequate support. It may however be sized differently compared to traditional recommendations for comfort or aesthetic reasons.

**Fig. 6.**
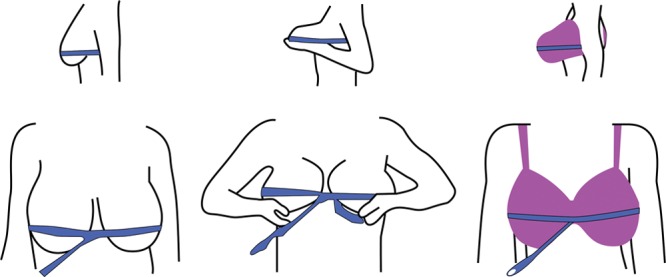
Three different ways of measuring overbust (ie, bust circumference) measurements, resulting in 3 entirely different measurements.

Breast morphology also varies in terms of chest wall positions and base diameters. Larger breasts tend to become more ptotic and bulbous, making accurate bust measurements increasingly difficult (Fig. 6).^8,11^ This suggests that the traditional technique in determining bra size can be very inaccurate, and the inherent error is compounded by many different styles of bras manufactured and the lack of standardization between brands. This is why to control for these variables, our study elicited the help of (1) a professional bra fitter, along with (2) bras from a single style and company only. If preoperative and expected postoperative bra sizes are elements to be considered as part of a breast reduction consultation, the accuracy of bra fit should be examined and regardless of the technique, measurements obtained should only act as a guide to determining bra size.^10^

Another potential limitation of this study is the relative subjectivity involved in filling the cups with modeling clay, which may be less compressible than breast tissue. The research group was greatly assisted by the availability of an experienced professional bra fitter. This expertise would not commonly be available in the clinical setting. Hence, it would be beneficial for the surgeon to have sound knowledge about bra fitting techniques or for patients to visit a professional bra fitter before their initial consultation. The authors’ experience in this project suggests that it would not be difficult for surgeons working in this area to either develop the necessary expertise or refer patients to selected commercial bra fitters.

It should be emphasized that this study is only a guide to obtaining desired cup size, and patients must be aware preoperatively that a particular cup size cannot be guaranteed or promised.

Future directions and studies to further guide resection volumes for cup sizes to supplement these data would be to analyze prospective data on how the patient’s actual bra cup sizes change with predicted compared with actual data. Additionally, a further arm would be to compare to 3-dimensional imaging volume assessments to our weight assessments for reduction mammaplasty. Three-dimensional imaging has been shown to be most reproducible for actual breast volume assessments and prediction in breast augmentation surgery.^13,14^

## CONCLUSION

A table was derived from this study showing the weight of breast tissue to excise in reduction mammaplasty to achieve the desired cup size. This can be used as a reference guide for resection volume in breast reduction procedures or as a secondary use, volume estimates for implant sizing. This guide should be used in conjunction with adequate consultation and thorough understanding between the surgeon and patient in regard to the definition of the preoperative breast size and the expectation of the postoperative breast size.

## ACKNOWLEDGMENTS

The authors would like to thank Eastern Health Surgical Research Group, Department of Surgery, Monash University, Eastern Health, Melbourne, VIC, Australia; Michael Lo and Adel Morsi, Plastic Surgery Unit, Eastern Health, Melbourne, VIC, Australia; Richard Masters, General Surgery (Breast) Unit, Box Hill Hospital, Eastern Health, Melbourne, VIC, Australia; Sue MacDonald, Triumph International (Australia) Pty Ltd, Southbank, VIC, Australia. This study was approved by the Eastern Health Research and Ethics Committee.
